# Is Age a Determinant Factor in EVAR as a Predictor of Outcomes or in the Selection Procedure? Our Experience

**DOI:** 10.5935/1678-9741.20160037

**Published:** 2016

**Authors:** Rui Machado, Gabriela Teixeira, Pedro Oliveira, Luís Loureiro, Carlos Pereira, Rui Almeida

**Affiliations:** 1 Hospital de Santo António - Centro Hospitalar do Porto, Porto, Portugal; 2 Instituto de Ciências Biomédicas Abel Salazar (ICBAS), Porto, Portugal

**Keywords:** Age Groups, Aortic Aneurysm, Abdominal, Endovascular Procedures

## Abstract

**Introduction:**

Endovascular aneurysm repair (EVAR) is the therapy of choice in high risk patients with abdominal aortic aneurysm. The good results described are leading to the broadening of clinical indications to younger patients. However, reintervention rates seem higher and even with successful treatment sometimes there is growth of the aneurysm sac and rupture, meaning a failure of the therapeutic goal. This study proposes to analyse the impact of age in patients' selection and post-EVAR results.

**Methods:**

The clinical records of consecutive patients undergoing endovascular aneurysm repair, between 2001 and 2013, were retrospectively reviewed. Patients were divided according to age groups (<70, 70-80 and >80 years). Gender, body mass index, aneurysm anatomic features, neck characteristics, iliac morphology, surgical indication, endograft type, anesthesic risk classification, length of stay, reinterventions and mortality were analysed and compared.

**Results:**

The study included 171 patients, 161 (94.1%) men, and mean age 74.1±8.9 years. The age group under 70 had 32% of the patients. Only three characteristics were found different among age groups: 1) body mass index was higher in younger patients, with a considerable trend toward significance (P=0.06); 2) surgical indication, in the younger group, surgeon's and the patient's option were more proeminent (*P*<0.05); 3) erectile dysfunction was higher in elderly group (*P*<0.05). No other clinical and anatomical characteristics or final outcomes were found statisticaly different among age groups.

**Conclusion:**

The absence of statistically differences in mortality and reinterventions among age groups suggests that age by itself is not a relevant factor in endovascular aneurysm repair. Indeed, the three characteristics different in younger (obesity, sexual function and patient's choice) favor endovascular aneurysm repair.

**Table:** 

Abbreviations, acronyms & symbols	
AAA	= Abdominal aortic aneurysm
ASA	= American Society of Anesthesiologists
BMI	= Body mass index
EVAR	= Endovascular aneurysm repair

## INTRODUCTION

Since Parodi's publication in 1991 about the first endovascular aneurysm repair (EVAR)^[1]^, this therapy has been widely used in elderly and high-risk patients. Several randomized controlled trials showed less mortality, a shorter length of stay, lower consumption of blood products, and a better short-term quality of life with EVAR^[2,5]^. When it comes to younger patients, it is imperative to question the procedure's durability, reintervention rates, and the probability of aneurysm sac growth and rupture. The purpose of this study is to investigate if there is any association of age during EVAR, clinical characteristics of the patients and the outcomes of the procedure compared among different age groups.

## METHODS

The clinical records of consecutive patients undergoing EVAR for infrarenal abdominal aortic aneurysm (AAA) or aortoiliac aneurysms between October 2001 and December 2013 in our department were retrospectively reviewed. All surgeries took place in an operation room equipped with a Philips BV 300 C-Arm and a radiolucent table, and were performed by the same surgical team. The surgical criteria were infra-renal fusiforme AAA with diameter equal or superior to 5 cm and AAA associated with iliac aneurysms with diameter equal or superior to 3 cm, saccular aneurysms and false aneurysms. The endografts used were Talent Bi- and Uni-Iliac (suprarenal fixation, by friction) and Gore-Excluder Bi-Iliac (infrarenal fixation, by barbs); after October 2008 the Endurant Bi- and Uni-Iliac (suprarenal fixation, by hooks) were used. Patients were divided by age. The groups were: patients younger than 70 years old, 70 to 80 years old and older. Sex, atherosclerotic risk factors (hypertension, diabetes, smoke history, dyslipidemia), cerebrovascular disease, peripheral arterial disease, body mass index (BMI), anatomic features, aneurysm diameter, neck characteristics (diameter, angulation, length, calcification, thrombus), iliac morphology (tortuosity and diameter), anatomical risks, internal iliac artery aneurysm, surgical indication for EVAR, endograft type, American Society of Anesthesiologists (ASA) classification, anesthetic technique, length of stay, re-interventions, mortality, and costs were analyzed and compared.

### Statistical Analysis

Statistical analysis included t-tests for two independent samples, analyses of variance in the case of several groups, and chi-square tests for the comparison of categorical variables. Nonparametric tests were used when the normality or homogeneity of variances was not observed. All the analyses were performed using IBM SPSS Statistics, version 22; the statistical significance of two-sided tests was assumed to be *P*<0.05.

## RESULTS

One hundred and seventy-one patients, of which 94.2% (161/171) were men, underwent EVAR. The mean age was 74.1 years and the median was 75, with a standard deviation of 8.9 (min.: 38; max.: 93). The under-70 age group had 32% of the patients, 38.4% were between 70 and 80 years old, and 29.7% were more than 80 years old. The median time of follow-up was 32.7±29.8 months.

Pre-, intra-, and postoperative data are shown in Table 1. Atherosclerotic risk factors, cerebrovascular and peripheral arterial disease and BMI are shown in Table 2. Younger patients had a higher BMI, near to statistical significance (*P*=0.06).

**Table 1:** Pre, intra and postoperative data. No statistical significance. IIA, Internal Iliac Artery; CIA, Common Iliac Artery; ASA, American Society of Anaesthesiologists classification.

	< 70 years																																																												< 70 years																																																												< 70 years																																																											
Aneurysm Morphology	Aortic															Right AortoIliac															Left AortoIliac															Bilateral AortoIliac															Aortic															Right AortoIliac															Left AortoIliac															Bilateral AortoIliac															Aortic															Right AortoIliac															Left AortoIliac															Bilateral AortoIliac														
	30.4%															34.6%															38.5%															30%															41.1%															30.8%															53.8%															45%															28.6%															34.6%															7.7%															45%														
IIA Aneurysm	5.8%																																																												12.1%																																																												12%																																																											
Aneurysm Diameter	61.7mm (min 27 ; max 104)																																																												61.2mm (min 30 ; max 106)																																																												64.5 (min 25 ; max 103)																																																											
Neck Diameter	22mm (min 16 ; max 30)																																																												22.8 (min 18 ; max 33)																																																												22.8mm (min 16 ; max 32)																																																											
Neck Length	22.5 (min 10 ; max 70)																																																												22.9mm (min 10 ; max 80)																																																												23.6mm (min 6 ; max 50)																																																											
Neck Shape	Straight															Conical															Inverted															Other															Straight															Conical															Inverted															Other															Straight															Conical															Inverted															Other														
	62%															28%															6%															4%															61.4%															29.8%															3.5%															5.3%															69.8%															26.5%															4.7%															0%														
Calcification	<50%																				>50%																				None																				<50%																				>50%																				None																				<50%																				>50%																				None																			
	75%																				25%																				0%																				68.3%																				26.7%																				5%																				64.3%																				26.2%																				4.5%																			
Thrombus	None												<25%												25-50%												50-75%												>75%												None												<25%												25-50%												50-75%												>75%												None												<25%												25-50%												50-75%												>75%											
	79.2%												2.1%												6.3%												10.4%												2.1%												63.8%												6.9%												10.3%												17.2%												1.7%												70.7%												4.9%												12.2%												7.3%												4.9%											
Angulation	None																				<50°																				>50°																				None																				<50°																				>50°																				None																				<50°																				>50°																			
	24.5%																				42.9%																				32.7%																				18.6%																				47.5%																				33.9%																				9.5%																				52.4%																				38.1%																			
Iliac Tortuosity	Small																				Medium																				Large																				Small																				Medium																				Large																				Small																				Medium																				Large																			
	43.2%																				37.8%																				18.9%																				33.3%																				44.4%																				22.2%																				19%																				38.1%																				42.9%																			
Right CIA Diameter	<20mm																														>20mm																														<20mm																														>20mm																														<20mm																														>20mm																													
	85.7%																														14.3%																														78.9%																														21.1%																														83.3%																														17.7%																													
Left CIA Diameter	<20mm																														>20mm																														<20mm																														>20mm																														<20mm																														>20mm																													
	72%																														28%																														78.9%																														21.1%																														83.3%																														17.7%																													
ASA	II																				III																				IV																				II																				III																				IV																				II																				III																				IV																			
	22.6%																				64.2%																				13.2%																				16.1%																				69.4%																				14.5%																				6.4%																				80.9%																				12.7%																			
Anaesthesia	General																				Local																				Loco-regional																				General																				Local																				Loco-regional																				General																				Local																				Loco-regional																			
	38%																				0%																				62%																				42.2%																				4.7%																				53.1%																				23.4%																				6.4%																				70.2%																			
Length of Stay	7.2 days (min 2 ; max 60)																																																												6.17 days (min 1 ; max 40)																																																												5.54 days (min 2 ; max 15)																																																											
Re-Intervention	9.3%																																																												21.2%																																																												17.6%																																																											
30-day Mortality	0%																																																												0%																																																												3.9%																																																											

**Table 2:** Atherosclerotic risk factors, cerebrovascular disease, peripheral arterial disease and body mass index (kg/m^2^), by age group.

		Age groups			
		< 70 years	70-80 years	> 80 years	SS
Hypertension		83.3%	90.9%	76.5%	N
Active smokers		38.9%	7.7%	3.9%	N
Former smokers		48.1%	70.8%	54.9%	N
Dyslipidemia		66.7%	73.8%	60.8%	N
Diabetes		20.4%	13.8%	21.6%	N
Cerebrovascular disease		24.1%	26.2%	5.9%	N
Peripheral arterial disease		18.5%	21.5%	13.7%	N
Atherosclerotic risk factors association	No risk factors	25%	25%	25%	N
	1-2 risk factors	32.6%	30.2%	37.2%	
	3-4 risk factors	30.9%	41.5%	27.7%	
	5 or + risk factors	38.9%	55.6%	5.6%	
BMI	< 25 kg/m^2^	30.6%	34%	60.4%	*P*=0.06
	25-30 kg/m^2^	38.8%	41.5%	33%	
	> 30 kg/m^2^	30.6%	24.5%	17.7%	

SS =statistical significance; Y=yes; N=no

**Fig. 1:**
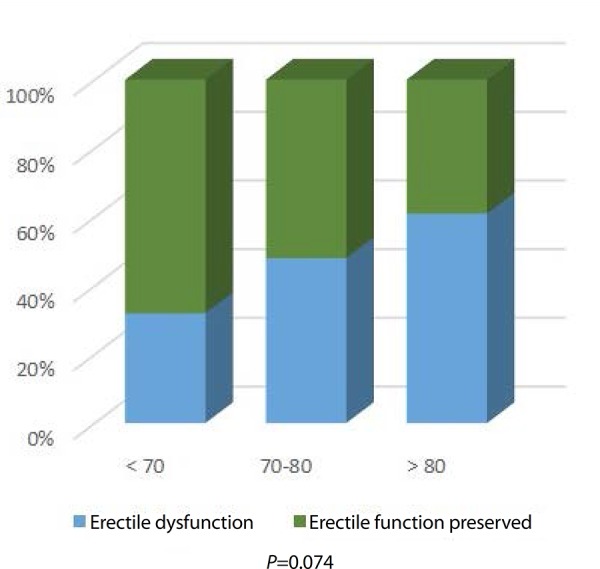
Pre-EVAR erectile dysfunction by age group; P=0.074.

**Fig. 2:**
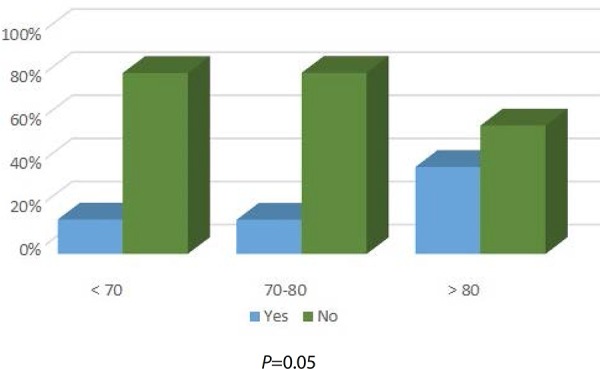
Blood transfusion during hospitalization by age group, P=0.04.

Regarding erectile function, 44.6% patients, with mean age of 75.5±7.2 years, presented dysfunction pre-EVAR, compared to 55.4% patients with erectile function preserved, with a mean age of 70±8.7 years. This age difference was statistically significant (*P*<0.05). Grouping erectile dysfunction by age, there is an obvious tendency for it to increase with age (Figure 1). The need for blood transfusion during hospitalization was also higher in older patients, as shown in Figure 2, and this difference was statistically significant (*P*<0.05).

Indications for EVAR were divided in a high-risk profile (clinical + hostile abdomen), surgeon's option, and patient's option. In the under-70 age group, the decisions were 73.6%, 15.1%, and 11.3%, respectively; in the 70-80 age group, they were 80%, 12.3%, and 7.7%, respectively, and in the age >80 group they were 98%, 2%, and 0%, respectively. There was a statistically significant difference among the age groups and indication for EVAR: in the younger group, the surgeons' and the patients' options were more frequent. The types of endografts used are described in Table 3.

**Table 3:** Type of endograft used.

	< 70 years					70-80 years					> 80 years				
Endograft	Excluder	Endurant		Talent		Excluder	Endurant		Talent		Excluder	Endurant		Talent	
		Bi-Iliac	Uni-Iliac	Bi-Iliac	Uni-Iliac		Bi-Iliac	Uni-Iliac	Bi-Iliac	Uni-Iliac		Bi-Iliac	Uni-Iliac	Bi-Iliac	Uni-Iliac
	42.59%	25.93%	16.6%	1.85%	7.41%	36.29%	32.31%	21.54%	4.62%	4.62%	24.49%	57%	22.45%	6.12%	14.29%

No statistical significance.

**Fig. 3:**
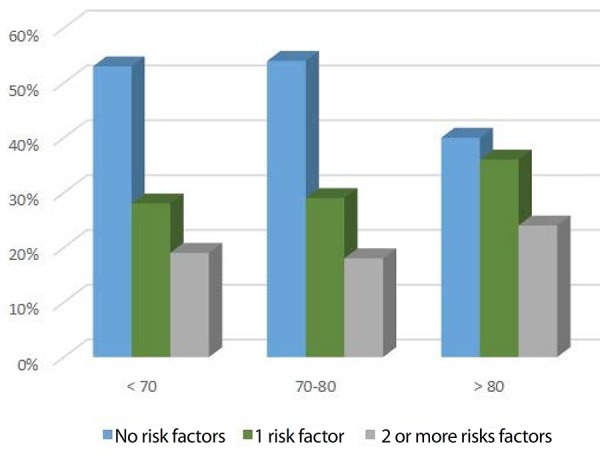
EVAR risk factors association by age group; no statistical significance.

Anatomic risk factors for EVAR were defined as a neck angle >50º, a neck diameter >28 mm, a neck length <10 mm, calcification of >50% of the neck circumference, thrombus >50% of the neck circumference, iliac diameter >20 mm, and iliac tortuosity. In Figure 3, we divided the number of risk factors presented into 0, 1, and 2 or more risk factors by age groups. No significant relationship was found between risk factors and age groups.

We reported no statistical difference in the incidence and type of endoleak, as shown in Tables 4 and 5, or in aneurysmal sac behavior (Table 6).

**Table 4:** Presence of endoleak by age group.

Age group	No endoleak	With endoleak
<70 years	63%	37%
70-80 years	56.3%	43.7%
>80 years	59.6%	40.4%

No statistical significance.

**Table 5:** Type of endoleak by age group.

Age Group	No endoleak	Endoleak I or III	Endoleak II	Endoleak II + I/III
<70 years	63%	3.7%	25.9%	7.4%
70-80 years	56.3%	12.5%	28.1%	3.1%
>80 years	59.6%	12.8%	19.1%	8.5%

No statistical significance.

**Table 6:** Aneuysmal sac behavior after EVAR

	<70 years	70-80 years	>80 years
Sac growth	8.0%	11.3%	13.3%
Sac shrinkage			
0-5 mm	26.0%	16.1%	24.4%
5-10 mm	30.0%	32.3%	33.3%
10-15 mm	6.0%	14.5%	17.8%
15-20 mm	8.0%	14.5%	4.4%
20-25 mm	8.0%	6.5%	2.2%
25-30 mm	8.0%	1.6%	2.2%
>30 mm	6.0%	3.2%	2.2%

No statistical significance.

Two deaths were registered on the 30^th^ day of the follow-up period, both in the >80 age group. The global mortality rate was 1.2% (2/171).

**Fig. 4:**
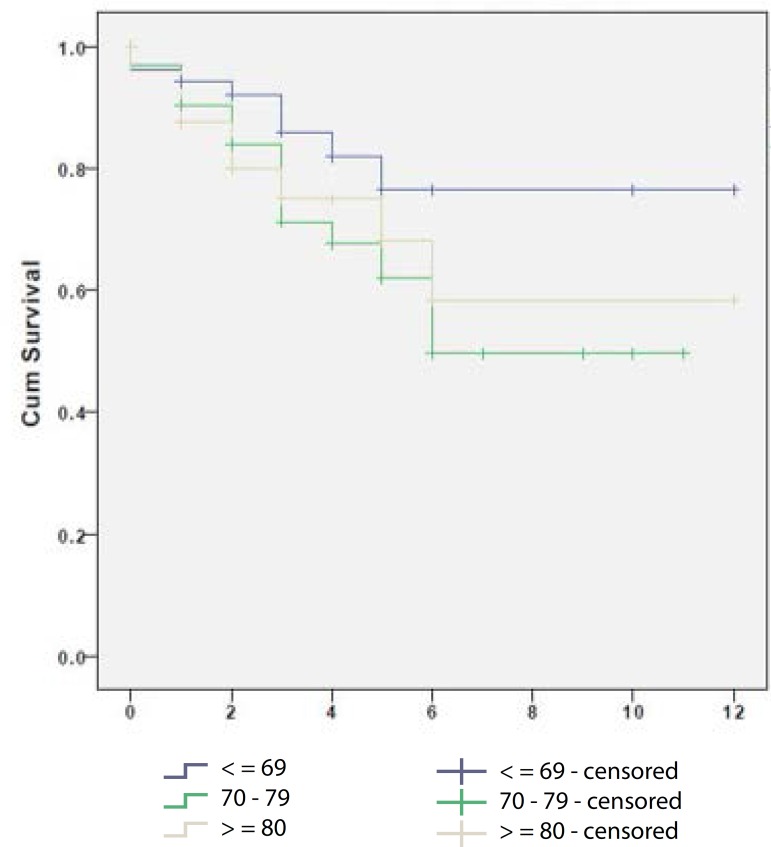
In the population below 70 years, the median survival were 9.9 years, standard deviation 0.7 (CI 95 - 8.5 to 11.2). The population with 70-80 years had a median survival of 7.2 years, standard deviation 0.7 (CI 95 - 5.8 to 8.6). The patients older than 80 years had a median survival of 8.3 years, standard deviation 1 (CI 95 - 6.4 to 10.3).

Figure 4 presents 12-year cumulative survival after EVAR. Estimating the effect of age, we found that survival in the youngest age group (>70 years) was higher but with no statistical significance.

The mean procedure cost for patients with <70 years was 11.658€, for patients between 70-80 years was 11.110,3€, and for patients with >80 years, the cost was 11.521,8€ (Table 7).

**Table 7:** Mean operative costs for patients undergoing EVAR.

		Mean	Median	Std. Deviation	Minimum	Maximum
EVAR	<70 years	11,658 €	10,226.80 €	6,157.80 €	9,270 €	50,779.70 €
	70-80 years	11,110.30 €	10,484.70 €	2,163 €	9,433 €	20,979 €
	>80 years	11,521.80 €	10,371.30 €	4,752.80 €	9,081.50 €	40,240.70 €

No statistical significance.

## DISCUSSION

It is the consensus that EVAR is indicated in elderly and highrisk patients. However, in light of good results, the indication for EVAR has been progressively extended to younger and less highrisk patients, and also to patients with added anatomical risks. So, it is fair to question if age is an important factor in choosing EVAR as the best therapeutic choice.

EVAR growth has been overwhelming. Albuquerque et al.^[6]^ documented a significant increase in the number of EVARs performed between 2005 and 2008, with an average rate of 84%, compared to a rate of 42.2% between 1996 and 2002. Schwarze et al.^[7]^ reported a 162% increase for patients >85 years old between 2001 and 2006. For those younger (50-64 years old), the increase was less pronounced. Still, in 2005 and 2006, for this age group EVAR was already a commonly chosen procedure.

A factor to consider when choosing a therapy is the life expectancy of this group of patients. Altaf et al.^[8]^ mentioned that independently of the technique used in AAA treatment, in young patients, the mortality rate in 6 years was 40%, with most of the deaths unrelated to the aneurysm. Darwood et al.^[9]^ stated that the group of patients with aneurysms 4-5.4 cm in diameter diagnosed by a screening in Gloucestershire had a 10-year mortality of 58%. Therefore, the survival rate of the patient with an AAA can be much lower than that of the general population.

Mani et al.^[10]^, using the Swedish Vascular Registry from 1987 to 2005, published a 5-year survival rate of 69% and a 10-year survival rate of 39.3% after elective correction of AAAs. Mean survival was 8.9 years (99% CI: 8.7-9.2). No significant difference in relative survival was observed among different age groups. In our study, we estimated survival curves of 9.9 years for those younger than 70 years, 7.2 years for those aged 70-80 years, and 8.3 years for the elderly, those over 80 years old.

The published randomized trials EVAR-1^[2,11]^, DREAM^[3]^, and OVER^[4]^, comparing EVAR with conventional surgery, showed that early death rates were lower with EVAR (1.7% vs. 4.7%, P=0.009; 1.2% vs. 4.6%, P=0.10; and 0.5% vs. 3%, P=0.004, respectively). EVAR-1^[11]^ showed higher reintervention rates, all graft related (23.2% vs. 8.9%, *P*<0.001). The OVER study included laparotomy-related complications (incisional hernia, bowel ischemia or obstruction) and showed similar reintervention rates (22.1% vs. 17.8%, *P*=0.12); the time to a secondary therapeutic procedure or death was also similar (1.06 years, *P*=0.57).

The ACE study showed a death rate higher with EVAR compared to conventional surgery (1.6% vs. 0.6%, P=0.09, not statistically significant) and a higher reintervention rate (16% vs. 2.7%, *P*<0.0001), but reinterventions for incisional repair were not recorded^[5]^.

In our study, a global mortality of 1.2% (very similar to the mortality rates published in the literature after EVAR) was noted. By age group, zero mortality was noted in both the <70 and 70-80 age groups, and two deaths were registered in the >80 age group (2/51, 3.9%). Although not statistically significant, the trend to lower mortality suggested the safety of EVAR in younger people.

Reinterventions seem to be more frequent after EVAR, but the majority are minor endovascular reinterventions with relatively low 30-day mortality, as stated by Giles et al.^[12]^. Recently, Lee et al.^[13]^ described a nonsignificant difference in the reintervention rate between EVAR (16%) and conventional surgery (12%) in a single center retrospective review. In our study, there was no statistical difference in reinterventions among different age groups. Indeed, the number of reinterventions was smaller in younger groups: 9.3% (<70 years old), 21.2% (70-80 years old), and 17.6% (>80 years old).

The EVAR-2 study compared a set of patients unfit for open repair^[14]^. This study is the closest to our experience, as the majority of our patients submitted to EVAR had clinical contraindications for open surgery. In EVAR-2 this group was submitted to EVAR or underwent clinical follow-up. This study showed a higher death rate with EVAR compared to the EVAR-1 study (9% vs. 2.1%). However, Lim et al.^[15]^, applying the same criteria as EVAR-2, had mortality rates much lower and similar to the remaining literature.

The population study made by Schernerhorn et al.^[16]^ comparing EVAR with open repair revealed a mortality rate of 1.2% vs. 4.8%, respectively. The benefits of EVAR were still present after three years of follow-up, when both procedures' results were matched. In the 64-74-year-old age, the results are comparable after the first year, while in the group of patients over 85 years old there was still an advantage for the EVAR procedure in the fourth year.

The indication for EVAR in young patients can be questioned after Schanzer's et al.^[17]^ publication. This analysis revealed that 5 years after EVAR, there was aneurysm sac growth over 5 mm in 41% of patients. The predisposing factors to this growth were conical aortic neck, aortic neck diameter >28 mm, neck angle >60º, iliac diameter >20 mm, and the presence of an endoleak. As younger patients had a longer life expectancy, it seems to also present a higher risk of sac growth. However, in this study, the primary determinant of AAA sac enlargement was the presence of an endoleak, and the majority of endoleaks (76%) became evident during the first year post-EVAR. Besides, analyzing additional significant predictors of AAA sac enlargement in a multivariable analysis in those over 80 years old, age was also considered one predisposing risk factor. In our study, there was no significant difference in sac enlargement between older and younger patients.

In our study, considering the retirement age in the public sector (70 years) and life expectancy in Portugal (81 years, according to the Global Health Observatory Data Repository, 2012, http://apps.who.int/gho/data/node.main.688?lang=en), patients under 70 were considered young.

In contrast to randomized studies, with carefully selected patients, ours is a real-life study with all consecutive patients submitted to EVAR. There is a relevant difference in ASA physical status classification among our patients and others mentioned above. We can classify our population as ASA I (0%), ASA II (15.4%), ASA III (71%), and > ASA IV (13.6%), compared to 10.6%, 65.6%, 22.5%, and 1.3%, respectively, as reported by the ACE trials^[5]^ and 21.4%, 70.5%, 8.1%, and 0%, as described by the DREAM group^[18]^.

Therapeutic choice was based on a set of parameters such as the surgical risk (both clinical and anatomic), the anatomical characteristics of the aneurysm, and the surgeon's and patient's option. Winterborn et al.^[19]^, after a semistructured telephone interview with patients with small AAAs, concluded that the majority would prefer EVAR. Their major fears were the risk of organ failure and death. The type of incision, radiation exposure, and the risk of sexual dysfunction were ranked as the least important. Another three studies placed EVAR first among patients' preferences, for whom lower short-term morbidity and mortality and a shorter length of stay took precedence over a higher risk of future reinterventions^[19,21]^. In our study, the surgeons' and patients' preferences for EVAR were statistically relevant in the younger group.

Another factor was relevant among age groups, and it may have had an impact on therapeutic choice: erectile dysfunction was significantly more frequent in older patients. As described elsewhere, sexual function deterioration is more frequent after open surgery^[22]^. So, we can assume that for younger patients, with preserved sexual activity, EVAR could be a better solution, as long as the internal iliac artery is preserved, as it does not interfere with parasympathetic chains and has a lower risk of producing sexual dysfunction.

During hospitalization, older patients needed a great number of blood transfusions. This difference was statistically significant and may suggest lower rates of early complications in younger patients. We haven't observed different costs between different age groups.

Obesity is a known risk factor for open surgery, and it is associated with worse outcomes. As published in a meta-analysis, after a review of 4 studies with a total of 2440 patients, 30-day postoperative mortality was statistically higher with open surgery, as was myocardial infarction, chest infection, renal failure, and wound infection, compared to obese patients submitted to EVAR^[23]^. In our study, one-third of the younger group was obese and, in these patients, EVAR is favored over open surgery.

Veith et al.^[24]^, arguing against level 1 studies, had no doubts: in fit patients with suitable anatomy, and when performed with requisite skills, facilities, and equipment, "do it by endovascular aneurysm repair!". Their position is based on several points: first, we do it better than we did it when we started, so the actual results are better than those published 10 years ago, and we still considered level 1 evident (EVAR-1, OVER); second, endoprotheses are better, with lower migration and late failure and, finally, now we know and understand complications and we treat them in a useful time period (in EVAR-1, all endoleaks were considered complications and many complications were detected but left untreated). Also EVAR processing technical details have been improved, as recently stated by Molinari et al.^[25]^, who concluded that as time goes by, the level of performance has increased and interventional procedures are done more efficiently, with less contrast injections and exposure to ionizing radiation.

## CONCLUSION

Analyzing our experience, the absence of statistical differences between mortality and reinterventions between different age groups reveals that age itself is not a relevant factor in EVAR outcomes. Indeed, the three characteristics statistically different in younger patients (obesity, sexual function, and patient's choice) can favour EVAR. No other clinical variables, surgical reintervention, mortality or costs were statistically different among age groups. Thus, we can suggest that age is not important as association to procedure-related EVAR outcomes when compared to older patients, and thereby may not be decisive in surgeon's choice towards EVAR in a younger population; however, the limitations of the retrospective analysis must be considered.

**Table:** 

Authors' roles & responsibilities	
RM	Conception and design study; operations and/or trials performance; analysis and/or data interpretation; manuscript writing or critical review of its content; final manuscript approval
GT	Manuscript writing or critical review of its content; final manuscript approval
PO	Statistical analysis; final manuscript approval
LL	Operations and/or trials performance; final manuscript approval
CP	Operations and/or trials performance; final manuscript approval
RA	Final manuscript approval
